# The stressed female brain: neuronal activity in the prelimbic but not infralimbic region of the medial prefrontal cortex suppresses learning after acute stress

**DOI:** 10.3389/fncir.2013.00198

**Published:** 2013-12-20

**Authors:** Lisa Y. Maeng, Tracey J. Shors

**Affiliations:** Behavioral and Systems Neuroscience, Department of Psychology, Center for Collaborative Neuroscience, Rutgers UniversityPiscataway, NJ, USA

**Keywords:** sex difference, eyeblink conditioning, medial prefrontal cortex, prelimbic cortex, infralimbic cortex, stress, basolateral amygdala, classical conditioning

## Abstract

Women are nearly twice as likely as men to suffer from anxiety and post-traumatic stress disorder (PTSD), indicating that many females are especially vulnerable to stressful life experience. A profound sex difference in the response to stress is also observed in laboratory animals. Acute exposure to an uncontrollable stressful event disrupts associative learning during classical eyeblink conditioning in female rats but enhances this same type of learning process in males. These sex differences in response to stress are dependent on neuronal activity in similar but also different brain regions. Neuronal activity in the basolateral nucleus of the amygdala (BLA) is necessary in both males and females. However, neuronal activity in the medial prefrontal cortex (mPFC) during the stressor is necessary to modify learning in females but not in males. The mPFC is often divided into its prelimbic (PL) and infralimbic (IL) subregions, which differ both in structure and function. Through its connections to the BLA, we hypothesized that neuronal activity within the PL, but not IL, during the stressor is necessary to suppress learning in females. To test this hypothesis, either the PL or IL of adult female rats was bilaterally inactivated with GABA_A_ agonist muscimol during acute inescapable swim stress. About 24 h later, all subjects were trained with classical eyeblink conditioning. Though stressed, females without neuronal activity in the PL learned well. In contrast, females with IL inactivation during the stressor did not learn well, behaving similarly to stressed vehicle-treated females. These data suggest that exposure to a stressful event critically engages the PL, but not IL, to disrupt associative learning in females. Together with previous studies, these data indicate that the PL communicates with the BLA to suppress learning after a stressful experience in females. This circuit may be similarly engaged in women who become cognitively impaired after stressful life events.

## INTRODUCTION

Stressful life events are often accompanied by disruptions in cognitive and emotional processes related to learning and memory. In humans, stressful experiences can induce or exacerbate the symptoms of stress-related mental illness including post-traumatic stress disorder (PTSD) and generalized anxiety disorder (Kendler et al., 1995, 1999; Kessler, 1997; Brown, 1998; Lupien and Lepage, 2001; Yehuda, 2002; O’Donnell et al., 2004; Shors, 2004; McEwen, 2005; Lupien et al., 2009). Stressful life experiences have a significant impact on mental health in women, who are twice as likely as men to suffer from these disorders (Nolen-Hoeksema and Girgus, 1994; Breslau et al., 1997; Foa and Street, 2001; Tolin and Foa, 2006; Nolen-Hoeksema, 2012). Despite the alarming statistics, relatively little is known about the brain circuits and mechanisms that mediate sex differences in the stress response. There is, however, considerable information about the mechanisms that modulate the stress response in males. For example, the medial prefrontal cortex (mPFC) is necessary for mediating the effects of controllability during stress (Amat et al., 2005), and it can exert inhibitory control over the amygdala (LeDoux, 2000; Rosenkranz and Grace, 2001; Sotres-Bayon et al., 2004; Likhtik et al., 2005; Hoover and Vertes, 2007). Other studies in humans indicate that blood flow to these structures (and presumably neuronal activity) is disrupted by stressful life events. For instance, humans expressing symptoms of PTSD exhibit hyperactivity in the amygdala with concomitant mPFC hypoactivity (Coffey et al., 1993; Bremner et al., 1997; Liberzon et al., 2003; Shin et al., 2005; Rauch et al., 2006; Etkin and Wager, 2007; Koenigs and Grafman, 2009; Lebron-Milad et al., 2012; Tang et al., 2012; Kong et al., 2013; Wang et al., 2013). It has been suggested that neuronal hyperactivity in the amygdala occurs when the mPFC releases its control over it, which is theoretically necessary for emotional regulation. This putative mechanism is supported by reports of increased functional connectivity between the mPFC and amygdala during extinction recall, a phenomenon which is impaired in people suffering from PTSD (Milad et al., 2009).

Functional connections between the amygdala and mPFC mediate some sex differences in behavior in humans and rodent models (Bangasser and Shors, 2010; Goldstein et al., 2010; Lebron-Milad et al., 2012). For example, exposure to acute uncontrollable stress impairs associative learning in female rats, while exposure to the same event enhances learning in males (Wood and Shors, 1998; Wood et al., 2001; Bangasser and Shors, 2007; Waddell et al., 2008; Maeng et al., 2010; Maeng and Shors, 2012). Neuronal activity within the mPFC is necessary to induce the suppression of learning in females but is of no consequence in males (Maeng et al., 2010). Although these data indicate that activity within the mPFC is necessary to suppress learning in females, activity in this region alone is not sufficient, as activation with the GABA_A_ receptor antagonist picrotoxin did not suppress learning in females. Moreover, the mPFC must communicate with the basolateral nucleus of the amygdala (BLA) to suppress learning in females. Using a disconnection procedure, we determined that severing the connections between the BLA and the mPFC on both sides of the brain prevented the stress effect on learning but severing them on one side did not. From these studies, we concluded that the BLA and the mPFC communicate with one another to suppress learning after stress in females (Maeng et al., 2010).

The mPFC is often divided into two subregions – the prelimbic (PL) and the infralimbic (IL) cortex. They have different projections, both efferent and afferent, as well as different functional outputs. The PL cortex has robust projections to the basolateral amygdala (McDonald et al., 1996; Vertes, 2004), which may contribute to its role in the expression of anxiety and conditioned fear (Jinks and McGregor, 1997; Vidal-Gonzalez et al., 2006; Blanco et al., 2009; Burgos-Robles et al., 2009; Sierra-Mercado et al., 2011). The circuit that mediates the expression of fear involves excitatory input from the PL to the BLA, which activates the central amygdala to enhance conditioned fear (Quirk et al., 2003; Sotres-Bayon et al., 2004; Correll et al., 2005; Likhtik et al., 2005; Sierra-Mercado et al., 2011). The IL cortex, on the other hand, induces a suppression of conditioned fear expression. Based on these data, we hypothesized that during the stressful event, excitatory neuronal activity within the PL but not the IL region of the mPFC is necessary to suppress associative learning in females.

## MATERIALS AND METHODS

### SUBJECTS

The adult female Sprague Dawley rats (90–120 days of age) used in this study were bred in a facility at Rutgers University. The estrous cycle was monitored each day to ensure that all females had a 4–5 day cycle passing through the stages of proestrus, estrus, diestrus 1, and diestrus 2. They were housed in groups of 3–4 until surgery. After surgery, the rats were singly housed in standard plastic “shoebox” home cages (45 cm long, 22 cm wide, and 23 cm high). The animals were maintained on *ad libitum* access to rat chow and water on a 12 h light and 12 h dark schedule. The current experiments were conducted with full compliance to the rules and regulations specified by the Public Health Service (PHS) Policy on Humane Care and Use of Laboratory Animals and the Guide for the Care and Use of Laboratory Animals.

### SURGERY

Animals were anesthetized with sodium pentobarbital (50 mg/kg) and received trace amounts of isoflurane throughout the surgery to maintain anesthetization for the entire duration of the procedure. After the scalp was shaved and scrubbed with betadine, an incision was made with a scalpel. Holes were then drilled into the skull at the proper coordinates (noted below) into which cannula tips were lowered and allowed to settle for 1 min. These holes were then covered with bone wax prior to applying dental cement to hold the cannulas in place. For the first experiment, cannulas were bilaterally implanted into the PL subregion of the mPFC. Guide cannula (Plastics One, Inc.) were placed in the following locations relative to bregma at a 15° angle: +2.90 mm AP, ±1.60 mm ML, and -2.80 mm DV from the surface of the brain. For the second experiment, bilateral cannula were implanted into the IL area, with the following coordinates at a 30° angle and relative to bregma: +2.80 mm AP, ±3.10 mm ML, and -3.80 mm from dura.

To assess the eyeblink response, we used electromyography (EMG), which measures muscle activity, around the eyelid. To measure EMG, insulated wires (attached to a headstage) were implanted through the periorbital muscles of the eyelid. Additional electrodes were implanted to administer stimulation to the eyelid, which served as the unconditioned stimulus (US). Acrylic dental cement was applied to the skull and anchored by skull screws to secure the headstage and cannulas in place. To prevent occlusion, obturators were placed into the cannulas after implantation.

### VAGINAL CYTOLOGY

After recovery from surgery, animals were assessed for phases of the estrous cycle by vaginal smears. Each day, a cotton-tipped applicator was dipped in sterile 0.9% saline and inserted into the vaginal canal. Vaginal cells were placed onto slides and stained with 1% toluidine for estrous phase identification. Estrus is characterized as dense clusters of non-nucleated blue cornified cells, and proestrus is evident by dark purple-stained nucleated epithelial cells. Diestrus 1 is identified by a combination of leukocytes and few cornified epithelial cells, and diestrus 2 by very sparse leukocyte and nucleated epithelial cell types. All animals began experimentation during the diestrus 2 phase because the stress effect on learning is most pronounced during this phase of the estrous cycle (Shors et al., 1998).

### DRUG MICROINFUSIONS

Animals were acclimated to the conditioning chamber for 60 min, after which they were transferred to another room. The obturators were removed, and injectors (with projections 1 mm past the guide cannula) were inserted into cannula. Groups of female rats were bilaterally infused with either 0.5 μl artificial cerebrospinal fluid (aCSF) vehicle or 0.5 μg (1 μg/μl) of γ-aminobutyric acid (GABA_A_) receptor agonist muscimol. Muscimol suppresses neuronal activity within the regions of interest for several hours (Martin, 1991; Majchrzak and Di Scala, 2000). At the dose used in the present study, the effects of muscimol will have dissipated prior to the start of training 24 h later. All infusions were administered at a rate of 0.125 μl/min for 4 min for a total infusion volume of 0.5 μl. After 2 min to allow for diffusion, the obturators were replaced.

### STRESS PROCEDURE

Immediately following the microinfusions of either aCSF or muscimol, animals in the stressed groups were taken into another room (a different context from conditioning and infusions) and were subjected to inescapable swim stress. The animals were placed into a round plastic container about 12″ in diameter, which had been filled with room temperature water (21–23°) to a height of 11″. The rats were in the water for 15 min, after which they were thoroughly dried with a towel and returned to their respective home cages. Animals that were in the unstressed groups were returned to their home cages after the infusions.

### CLASSICAL CONDITIONING

Training occurred one day (24 h) after the end of stressor exposure. The rats were trained with classical eyeblink conditioning using a delay paradigm. The conditioned stimulus (CS) was an 80 dB, 850 ms white noise. The unconditioned stimulus (US) commenced 750 ms after the onset of the CS and co-terminated with it. The US consisted of a 100 ms, 0.5 mA periorbital eyelid stimulation, which is sufficient to reliably elicit an eyeblink response. Eyeblinks were detected as significant changes in the magnitude of the electromyographic (EMG) activity recorded from the eyelid muscles. To be considered a conditioned response (CR), the elevated EMG activity had to persist more than 10 ms and exceed 0.3 mV with a standard deviation of 3, when compared to the baseline activity recorded for 250 ms before the onset of each CS. Once the number of eyeblink responses was determined, the number of blinks that occurred 250 ms before the onset of the US was calculated. These responses are considered adaptive CRs because they occur close to the onset of the US and are not sensitized responses to the CS. Animals were exposed to 100 trials of training each day for 4 days. At the end of each day (session) of training, rats were returned to their home cages.

### STATISTICAL ANALYSIS

To assess performance, we calculated the percentage of CRs that were emitted over each of the four sessions (100 trials) of training. The percentage of responses was analyzed with stress versus no stress and drug versus vehicle as independent variables, with a within-subjects variable for sessions (days of training). Much of the acquisition occurs during the first 100 trials of training. To further assess differences in acquisition, the first 100 trials were analyzed in blocks of 20 trials. These data were analyzed with a mixed factor ANOVA. If the interactions were significant, Tukey HSD *post hoc* comparisons were used to detect significant differences between groups and variables.

### HISTOLOGY

To verify the location of the cannula, rats were injected intraperitoneally with a lethal dose of sodium pentobarbital (100 mg/kg) and transcardially perfused with 0.9% saline solution for exsanguination. This was followed by 10% buffered formalin. The obturators were replaced with injectors connected to 10 μl Hamilton syringes to infuse 0.5 μl Evans blue dye (1 mg/ml) to mark the site of infusion. Brains were then removed and post-fixed in formalin for at least 24 h. The brains were transferred from the 10% buffered formalin to a 30% sucrose-formalin solution for at least three days for cryoprotection. When the brains were fully saturated, they were frozen and sectioned into 40 μm thick coronal sections using a cryostat. Every third slice was mounted onto pre-gelled slides and stained with 0.1% neutral red to verify the accuracy of cannula placements.

**FIGURE 1:**
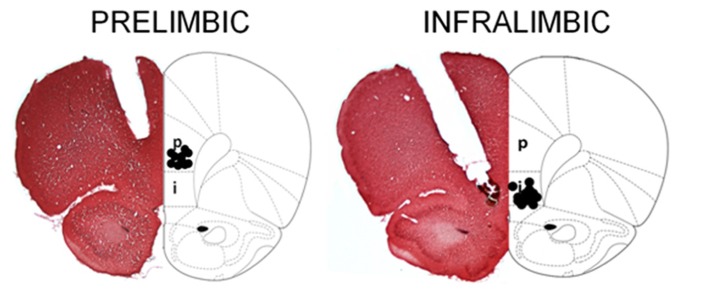
**Representative sections were stained with 0.1% neutral red, and reconstructions of the bilateral cannula tip placement within the PL and IL subregions at bregma +3.20 mm are illustrated here.** For the PL, cannula tips were implanted at an angle of 15° to avoid damage to the sinus. For the IL, cannula tips were angled at 30° to avoid damage to the overlying prelimbic cortex. Animals whose infusion sites were not correctly placed were excluded from the study. It is noted that cortical damage due to the permanent cannula implantation appears extensive. However, animals that received aCSF infusions learned well, and therefore, the damage did not interfere with performance of the associative learning task [image adapted from Paxinos and Watson (1997)].

A rater blind to group assignments in the behavioral data assessed cannula tip locations. If the tip of the injection cannula, which protruded 0.5 mm beyond the guide cannula, was within the dorsal boundary of the PL cortex, then it was considered to be in the correct location for PL infusions. For IL infusions, the cannula tip sites needed to be within the IL region, leaving the PL area intact. Placements within the mPFC were between +3.20 and +2.70 mm relative to bregma. The sites of drug infusion were assessed by track markings of the infusion cannula (**Figure 1**). Rats were excluded from analysis if placements were not within either the PL or IL area, or if the mPFC was excessively damaged by the cannula or the infusions. In experiment 1, the number of animals in each group was as follows: vehicle aCSF and unstressed (*n* = 7), vehicle aCSF and stressed (*n* = 6), muscimol and unstressed (*n* = 10), and muscimol and stressed (*n* = 8). For experiment 2, the number of animals per group was: vehicle aCSF and unstressed (*n* = 8), vehicle aCSF and stressed (*n* = 7), muscimol and unstressed (*n* = 7), and muscimol and stressed (*n* = 7).

## RESULTS

### EXPERIMENT 1. NEURONAL ACTIVITY IN THE PRELIMBIC CORTEX IS NECESSARY TO SUPPRESS LEARNING AFTER STRESS IN FEMALES

Experiment 1 determined whether neuronal activity within the PL area of the mPFC was necessary for the stress-induced impairment of eyeblink conditioning in females. To test this, the PL cortex of adult female rats was bilaterally infused with either muscimol or aCSF vehicle in a different context from training or the stress procedure. Immediately following infusions, animals were taken into another room and were either stressed or unstressed. One day after stressor exposure, all rats were trained with 100 trials of delay eyeblink conditioning for four consecutive days.

**FIGURE 2:**
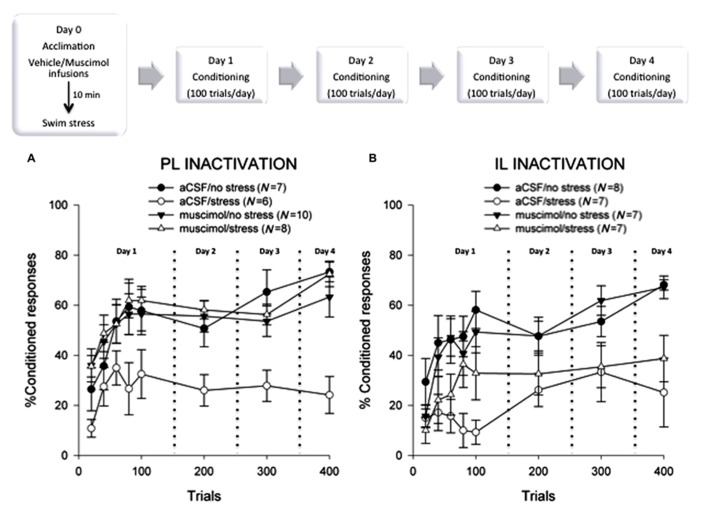
**(A)** Female rats that were injected with vehicle and exposed to acute swim stress 24 h before the 4 days of training emitted significantly fewer conditioned responses than those that were not stressed and expressed no evidence of learning (*p* < 0.05). Females that were stressed while their prelimbic mPFC was inactivated learned well, expressing more conditioned responses than the stressed controls (*p* < 0.05). These data suggest that neural activity within the prelimbic subregion of the mPFC is necessary to suppress learning in females after an acute stressful event. **(B)** Again, females that were injected with vehicle and exposed to acute swim stress emitted significantly fewer conditioned responses than those that were not stressed (*p* < 0.05). Likewise, those that were stressed while their infralimbic cortex was inactivated also did not learn (*p* > 0.05), expressing fewer conditioned responses than the unstressed animals (*p* < 0.05). These data suggest that neural activity within the infralimbic subregion of the mPFC is not part of the necessary circuit that impairs learning in females after a stressful event.

A 2 × 2 × 4 (stress versus no stress × drug versus vehicle × sessions of training) analysis of variance revealed a significant interaction between the injection with the GABA_A_-receptor agonist and stressor exposure [*F*(1,27) = 13.12; *p* < 0.01] and a significant three-way interaction among the agonist, stressor exposure, and sessions of training [*F*(3,81) = 2.75; *p* < 0.05]. A Tukey *post hoc* test revealed that the females that received bilateral aCSF injections into the PL before the stressor expressed fewer CRs than those that were not stressed (*p* < 0.01). Interestingly, females that were infused with muscimol into the PL cortex during the stressor emitted more CRs than those that were injected with the vehicle during the stressor (*p* < 0.01). As expected, the percentage of CRs increased across the four days of delay conditioning [*F*(3,87) = 6.68; *p* < 0.01], indicating that learning occurred over the sessions of training. A one-way repeated measures ANOVA indicated that stressed females injected with vehicle did not learn as they did not express more CRs across the four days of training [*F*(3,15) = 0.16; *p* > 0.05]. As illustrated in **Figure 2**, unstressed rats injected with muscimol 24 h before training performed similarly to their respective control animals that learned well (*p* > 0.05). Therefore, muscimol alone did not adversely affect conditioned responding 24 h after it was injected. It should also be noted that although there was cortical damage caused by the cannulation, it did not disrupt performance. The vehicle-injected control (aCSF/no stress) animals emitted consistent and well-timed CRs, which increased in number across sessions of training. It is also important to note that the effects of stress on performance of the CR are not due to performance effects, at least as far as can be proven. In previous studies, we found no effect of the stressor exposure on amplitude of the unconditioned response or on responses that are already acquired (Wood and Shors, 1998; Bangasser and Shors, 2004).

To examine early acquisition, the first session of 100 trials was analyzed as 20-trial blocks with a 2 × 2 repeated measures ANOVA. The analysis revealed a main effect of the agonist [*F*(1,27) = 4.42; *p* < 0.05] and block [*F*(4,108) = 20.90; *p* < 0.01]. Again, females infused with vehicle aCSF and stressed did not express many CRs during the first 100 trials, suggesting that they were unable to learn the association in response to the stressful event [*F*(4,20) = 2.76; *p* > 0.05].

**FIGURE 3:**
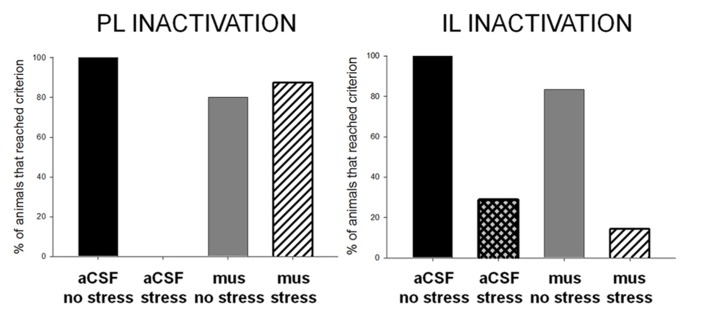
**Animals that learned the conditioned eyeblink response well reached a learning criterion of 60% conditioned responses.** Most or all of the unstressed vehicle- and muscimol-treated animals reached this learning criterion, whereas none or very few of the vehicle-treated stressed females did. Most of the females without activity in the prelimbic cortex during the stressor learned well, performing as well as females that were not stressed. However, very few of the females that were stressed while their infralimbic cortex was inactivated learned; they performed similarly to the stressed females without inactivation. These data support the conclusion that the prelimbic area of the mPFC, but not the infralimbic cortex, is critically engaged during a stressful event to suppress learning in females.

To further illustrate differences in performance, we have presented the data as the percentage of animals reaching a learning criterion of 60% conditioned responding in any session of training (**Figure 3**). The unstressed animals learned well, whereas all of the females that were exposed to the stressor and received vehicle did not. Interestingly, most (~88%) of the stressed females whose PL cortices were inactivated during the stressor learned well. The percentage of animals that reached the learning criterion differed between groups, χ^2^(1, *N* = 31) = 78.0, *p* = 0.00. Therefore, bilateral infusions of muscimol into the PL during the stressor prevented the effect of stress on conditioning. These data indicate that neuronal activity within the PL is necessary to suppress learning after stress in female rats.

### EXPERIMENT 2. ACTIVATION OF THE INFRALIMBIC CORTEX DURING THE STRESSOR IS NOT NECESSARY FOR THE STRESS EFFECT ON LEARNING IN FEMALES

Experiment 2 focused on the role of the IL subregion of the mPFC. The IL was bilaterally inactivated with muscimol infusions restricted to this region during the swim stressor. As before, learning was assessed with classical eyeblink conditioning 24 h after the stressor had ceased (**Figure 2**). A 2 × 2 × 4 analysis of variance for stress (no stress versus stress) × drug (aCSF versus muscimol) × training sessions revealed no effect of the GABA agonist [*F*(1,24) = 0.81; *p* > 0.05], but a main effect of stress [*F*(1,24) = 17.49; *p* < 0.01]. The percentage of CRs increased across sessions [*F*(3,72) = 6.69; *p* < 0.01], confirming that learning had occurred. Stressed females, however, that were infused with either muscimol [*F*(3,18) = 0.68; *p* > 0.05] or vehicle [*F*(3,18) = 0.91; *p* > 0.05] did not learn well as training progressed.

As in Experiment 1, we analyzed the percentage of CRs during each block of 20 trials on the first day of training. There was a main effect of stressor exposure [*F*(1,24) = 10.06; *p* < 0.01] and blocks of training [*F*(4,96) = 9.47; *p* < 0.01]. There was also a significant interaction between stressor exposure and the blocks of training [*F*(4,96) = 2.69; *p* < 0.05]. A Tukey *post hoc* analysis confirmed that the unstressed females that received either bilateral vehicle aCSF or muscimol injections into the IL emitted more CRs than the females that were injected with vehicle just before the stressor (*p* < 0.05). There was no deficit in responding as a result of the cannula implantation in general as unstressed, vehicle-treated rats could learn. Responding did not increase in the females that were injected with aCSF and stressed, when examined over the five 20-trial blocks of the first training session [*F*(4,24) = 1.11; *p* < 0.05]. The stressed, muscimol-treated females did emit CRs during the first day of training [*F*(4,24) = 3.02; *p* < 0.05], but performance was not maintained throughout the later sessions of training, as described above.

As in the first experiment, we analyzed data according to the number of animals that achieved a significant level of conditioned responding, which was 60% during at least one session of training. In the unstressed groups, most of the animals learned well, reaching at least 60% CRs in at least one session of training. In contrast, only 30% of the females that were infused with aCSF and stressed reached this learning criterion (**Figure 3**). Similarly, most of the animals that were stressed while their IL was inactivated did not learn (only 14% reached criterion). The percentage of animals that reached criterion did not differ between the intact or inactivated IL groups, χ^2^(1, *N* = 29) = 2.66, *p* = 0.10. These data further support the conclusion that neural activity within the IL during the stressor is *not* necessary to suppress associative learning in females.

## DISCUSSION

Exposure to an acute stressful event can dramatically impair new learning in females, when assessed with classical eyeblink conditioning (Wood and Shors, 1998). This effect depends on anatomical connections between the mPFC and basolateral amygdala, suggesting that the mPFC communicates with the amygdala during the stressful event to suppress learning in the near future (Maeng et al., 2010). The present set of experiments went beyond these findings to identify which part of the mPFC is involved – the PL or the IL. The PL cortex has dense projections to the basolateral amygdala, whereas the IL cortex does not. Based on these connections, we hypothesized that the PL region would be necessary. To test this hypothesis, each subregion was bilaterally inactivated during a short 15 min episode of inescapable swim stress. One day later, all animals were trained with classical eyeblink conditioning. Females with suppressed excitatory activity within the PL cortex during the stressor performed similarly to the unstressed females, rapidly learning the CR. In contrast, females with reduced activity in the IL cortex during the stressor behaved similarly to the stressed females with intact IL activity; neither group learned. These data suggest that the PL but not the IL cortex is critically engaged during a stressful event to suppress learning in females. Along with data from disconnection studies, we further conclude that the PL region of the mPFC communicates with the BLA during a stressful event to suppress learning in females.

### DIFFERENCES BETWEEN THE PRELIMBIC AND INFRALIMBIC CORTICES

There are numerous anatomical and functional differences between the PL and IL (Vertes, 2004; Izquierdo et al., 2006; Radley et al., 2006; Vidal-Gonzalez et al., 2006; Hoover and Vertes, 2007; Sierra-Mercado et al., 2011; Ball and Slane, 2012; Burgos-Robles et al., 2013). For instance, cells from the two regions fire with different patterns of activity during an operant conditioning task; PL neurons respond rapidly but transiently to reward, whereas IL neurons are slower to respond but respond for longer periods of time (Burgos-Robles et al., 2013). These data suggest a different time course of action between the IL and PL during the execution of the same behavior. Also, while immediate early gene c-fos activity increased within both regions immediately after exposure to a stressor, the response in the PL grew significantly larger with time (Bland et al., 2005). The present data are consistent with studies reporting that activity within the PL mPFC mediates the expression of conditioned fear (Choi et al., 2010, 2012; Sierra-Mercado et al., 2011; Sotres-Bayon et al., 2012; Pendyam et al., 2013). In fear conditioning, the PL augments conditioned fear responding. For instance, PL neuronal firing activity is greater in animals that fail to recall extinction, learning that requires the inhibition of conditioned fear responses. These animals express more conditioned fear (Burgos-Robles et al., 2009). In contrast, neuronal activity within the IL is critical for inhibiting conditioned fear after the animal has undergone extinction (Milad and Quirk, 2002; Lebrón et al., 2004; Vidal-Gonzalez et al., 2006; Sierra-Mercado et al., 2011). These regional differences in the mPFC may be important to the present phenomenon as well because excitatory activity within the PL during the 15 min swim stress was necessary to impair aversive conditioning, while activity within the IL was not. However, it remains possible that the longer lasting effects of stress on learning (those that occur after the stressor to maintain the suppression) could involve activity within the IL. Minimally, the present data suggest that activity within the PL and IL are differentially regulated by stressful life events to alter associative learning in females.

### PL CONNECTIONS TO THE AMYGDALA

**FIGURE 4:**
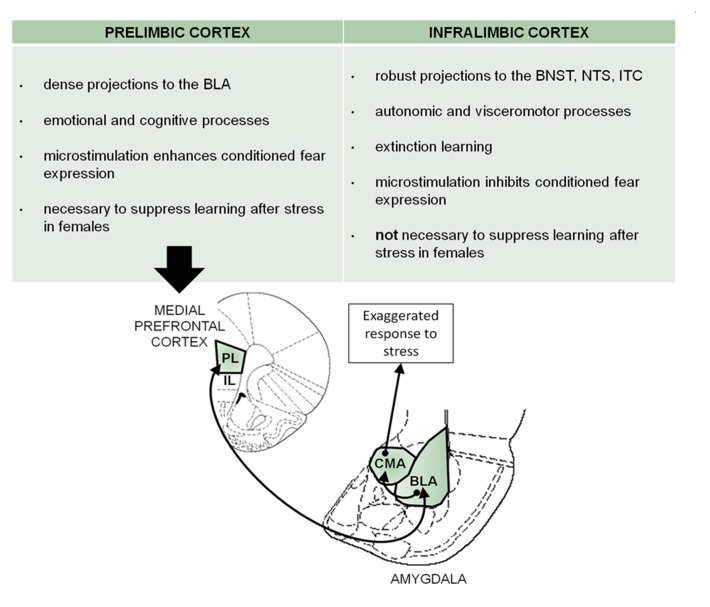
**There are both structural and functional differences between the prelimbic and infralimbic subregions of the mPFC.** Based on these differences and the present data, a prelimbic mPFC-BLA circuit in the modification of learning by stress is proposed here. In fear conditioning, this circuitry has been described for the expression of fear. Assuming that there is an overlap of circuitry with that mediating the responses to stress, we propose similar mPFC-BLA interactions that might modulate the stress-induced learning suppression in females. Prelimbic activity sends excitatory input to the basolateral amygdala, which stimulates the central amygdala to elicit the response to stress and impair conditioning [image adapted from Paxinos and Watson (1997)]. PL, prelimbic mPFC; IL, infralimbic mPFC; CMA, centromedial amygdala; BLA, basolateral amygdala; ITC, intercalated cells of the central amygdala; BNST, bed nucleus of the stria terminalis; NTS, nucleus of the solitary tract.

In humans, connections between the mPFC and amygdala have been implicated in mood disorders. Specifically, a hyperactive amygdala and hypoactive mPFC are associated with PTSD (Drevets, 2003; Liberzon et al., 2003; Rauch et al., 2006; Shin and Liberzon, 2010). These projections have been studied in animal models but with little attention given to the differentiation between the PL and IL subregions. The most extensive analysis of these mPFC regions has been conducted in laboratory animals as they express conditioned fear. The PL mPFC projects primarily to the BLA, whereas the IL has a wide distribution of projections (Vertes, 2004; Hurley et al., 1991). Interestingly, the PL cortex facilitates conditioned fear expression but does not appear to play a major role in conditioned fear inhibition, which relies on the IL. The identified circuit describes an excitatory input from the PL cortex to the BLA, which activates the central amygdala for the enhanced output of fear expression (Quirk et al., 2003; Sotres-Bayon et al., 2004; Correll et al., 2005; Likhtik et al., 2005; Sierra-Mercado et al., 2011). Based on the data presented here, it is possible that acute exposure to swim stress activates similar circuitry, which engages excitatory activity within the PL mPFC, which then communicates with the basolateral and centromedial (CMA) nuclei of the amygdala to suppress learning in females (**Figure 4**). Indeed, Maroun and Richter-Levin (2003) demonstrated that inescapable stress (30 min on a brightly lit elevated platform) impairs long-term potentiation in the mPFC induced by theta burst stimulation of the basolateral amygdala. Their recording electrodes were confined to the PL subregion. Therefore, these findings are consistent with those reported here, at least to the extent that they both implicate connections between the PL region and the basolateral amygdala as important in mediating responses to stressful life events.

### ESTROGEN IN THE AMYGDALA-mPFC CIRCUIT

Reproductive hormones, especially estrogen, alter the structure and function of the mPFC-amygdala circuitry. For instance, Shansky et al. (2010) reported that estrogen increased dendritic morphology of BLA-projecting neurons in the IL mPFC after chronic stress exposure. Dendritic remodeling may be associated with increased connectivity or function of the IL and the BLA. Similarly, chronic stress increases dendritic branching and length in the PL, which is mediated by activational effects of estradiol (Garrett and Wellman, 2009). Others have reported that stress-induced behaviors in females are mediated through activity within the dorsal mPFC, implicating PL but not IL regions (Calu et al., 2013). The effects of acute stress on classical conditioning presented here are dependent on the presence of estrogen, as they do not occur in ovariectomized females (Wood and Shors, 1998). They are also sensitive to naturally occurring changes in estrogen concentrations and are most evident when estrogen levels are rising. More specifically, the performance deficit is expressed when females are stressed during diestrus and trained during proestrus, as they begin to ovulate (Shors et al., 1998). We have also documented that exposure to an acute stressor decreases dendritic spine density in the hippocampus (Shors et al., 2001), although we did not investigate the mPFC. Based on these findings, it is proposed that exposure to the stressor induces morphological changes in BLA-projecting neurons of the PL cortex when in the presence of high circulating levels of estrogen. Such changes could thereby alter the neuronal circuits that are necessary to learn the association during classical conditioning.

The prefrontal cortico-amygdalar circuits are reportedly dysfunctional in women with major depressive disorder, whereas other circuits predominate in men (Kong et al., 2013). Increased activation of the amygdala and decreased activation of the prefrontal cortex were observed in women with PTSD as they anticipated the presentation of negative images. In contrast, women with fewer symptoms of PTSD presented more activity in their prefrontal cortex, which correlated with enhanced performance during attention shifting (Aupperle et al., 2012). These findings suggest that the prefrontal cortex and amygdala interact with one another in humans but are differentially responsive in women suffering from PTSD. The findings reported here may model some of the adverse consequences of stressful life experience in women, most notably acute stress disorder and PTSD. Alternatively, these responses may simply represent the “normal” healthy response to stressful life events.

## CONCLUSION

The present data indicate that the PL cortex is critically engaged during stress to impair learning in females, whereas the IL cortex is not. Based on previous data, we propose that acute stress exposure activates the prelimbic area and its connections to the basolateral amygdala. This may influence eyeblink circuitry in the cerebellum and/or hippocampus to elicit the learning deficit, an effect of stress that is only expressed in females. There is a higher prevalence of stress disorders in women, indicating a greater sensitivity to stressful life experience. The present data indicate that connections between the PL cortex and basolateral amygdala form part of the circuitry that mediates their vulnerability. This finding may have important implications for devising gender-considered therapies for women who suffer from stress-related illnesses.

## Conflict of Interest Statement

The authors declare that the research was conducted in the absence of any commercial or financial relationships that could be construed as a potential conflict of interest.

## References

[B1] Amat J. Baratta M. V. Paul E. Bland S. T. Watkins L. R. Maier S. F. (2005). Medial prefrontal cortex determines how stressor controllability affects behavior and dorsal raphe nucleus. *Nat. Neurosci.* 8 365–371 10.1038/nn1399 15696163 PMC13675003

[B2] Aupperle R. L. Allard C. B. Grimes E. M. Simmons A. N. Flagan T. Behrooznia M. (2012). Dorsolateral prefrontal cortex activation during emotional anticipation and neuropsychological performance in posttraumatic stress disorder. *Arch. Gen. Psychiatry* 69 360–371 10.1001/archgenpsychiatry.2011.1539 22474105

[B3] Ball K. T. Slane M. (2012). Differential involvement of prelimbic and infralimbic medial prefrontal cortex in discrete cue-induced reinstatement of 3, 4-methylenedioxymethamphetamine (MDMA; ecstasy) seeking in rats. *Psychopharmacology* 224 377–385 10.1007/s00213-012-2762-5 22710489 PMC4078904

[B4] Bangasser D. A. Shors T. J. (2004). Acute stress impairs trace eye blink conditioning in females without altering the unconditioned response. *Neurobiol. Learn. Mem.* 82 57–60 10.1016/j.nlm.2004.03.001 15183171 PMC3363961

[B5] Bangasser D. A. Shors T. J. (2007). The hippocampus is necessary for enhancements and impairments of learning following stress. *Nat. Neurosci.* 10 1401–1403 10.1038/nn1973 17906620 PMC3422868

[B6] Bangasser D. A. Shors T. J. (2010). Critical brain circuits at the intersection between stress and learning. *Neurosci. Biobehav. Rev.* 34 1223–1233 10.1016/j.neubiorev.2010.02.002 20153364 PMC2900534

[B7] Blanco E. Castilla-Ortega E. Miranda R. Begega A. Aguirre J. A. Arias J. L. (2009). Effects of medial prefrontal cortex lesions on anxiety-like behaviour in restrained and non-restrained rats. *Behav. Brain Res.* 201 338–342 10.1016/j.bbr.2009.03.001 19428654

[B8] Bland S. T. Schmid M. J. Der-Avakian A. Watkins L. R. Spencer R. L. Maier S. F. (2005). Expression of c-fos and BDNF mRNA in subregions of the prefrontal cortex of male and female rats after acute uncontrollable stress. *Brain Res.* 1051 90–99 10.1016/j.brainres.2005.05.065 15993862

[B9] Bremner J. D. Randall P. Vermetten E. Staib L. Bronen R. A. Mazure C. (1997). Magnetic resonance imaging-based measurement of hippocampal volume in posttraumatic stress disorder related to childhood physical and sexual abuse – a preliminary report. *Biol. Psychiatry* 41 23–32 10.1016/S0006-3223(96)00162-X 8988792 PMC3229101

[B10] Breslau N. Davis G. C. Andreski P. Peterson E. L. Schultz L. R. (1997). Sex differences in posttraumatic stress disorder. *Arch. Gen. Psychiatry* 54 1044 10.1001/archpsyc.1997.01830230082012 9366662

[B11] Brown G. W. (1998). Genetic and population perspectives on life events and depression. *Soc. Psychiatry Psychiatr. Epidemiol.* 33 363–372 10.1007/s001270050067 9708023

[B12] Burgos-Robles A. Bravo-Rivera H. Quirk G. J. (2013). Prelimbic and infralimbic neurons signal distinct aspects of appetitive instrumental behavior. *PLoS ONE* 8:e57575 10.1371/journal.pone.0057575 PMC3583875 23460877

[B13] Burgos-Robles A. Vidal-Gonzalez I. Quirk G. J. (2009). Sustained conditioned responses in prelimbic prefrontal neurons are correlated with fear expression and extinction failure. *J. Neurosci.* 29 8474–8482 10.1523/JNEUROSCI.0378-09.2009 19571138 PMC2733220

[B14] Calu D. J. Kawa A. B. Marchant N. J. Navarre B. M. Henderson M. J. Chen B. (2013). Optogenetic inhibition of dorsal medial prefrontal cortex attenuates stress-induced reinstatement of palatable food seeking in female rats. *J. Neurosci.* 33 214–226 10.1523/JNEUROSCI.2016-12.2013 23283335 PMC3711609

[B15] Choi D. C. Gourley S. L. Ressler K. J. (2012). Prelimbic BDNF and TrkB signaling regulates consolidation of both appetitive and aversive emotional learning. *Transl. Psychiatry* 2 e205 10.1038/tp.2012.128 PMC3565191 23250006

[B16] Choi D. C. Maguschak K. A. Ye K. Jang S. W. Myers K. M. Ressler K. J. (2010). Prelimbic cortical BDNF is required for memory of learned fear but not extinction or innate fear. *Proc. Natl. Acad. Sci. U.S.A.* 107 2675–2680 10.1073/pnas.0909359107 20133801 PMC2823921

[B17] Coffey C. E. Wilkinson W. E. Weiner R. D. Parashos I. A. Djang W. T. Webb M. C. (1993). Quantitative cerebral anatomy in depression. A controlled magnetic resonance imaging study. *Arch. Gen. Psychiatry* 50 7–16 10.1001/archpsyc.1993.01820130009002 8422224

[B18] Correll C. M. Rosenkranz J. A. Grace A. A. (2005). Chronic cold stress alters prefrontal cortical modulation of amygdala neuronal activity in rats. *Biol. Psychiatry* 58 382–391 10.1016/j.biopsych.2005.04.009 16023619

[B19] Drevets W. C. (2003). Neuroimaging abnormalities in the amygdala in mood disorders. *Ann. N. Y. Acad. Sci.* 985 420–444 10.1111/j.1749-6632.2003.tb07098.x 12724175

[B20] Etkin A. Wager T. D. (2007). Functional neuroimaging of anxiety: a meta-analysis of emotional processing in PTSD, social anxiety disorder, and specific phobia. *Am. J. Psychiatry* 24 202–218 10.1176/appi.ajp.2007.07030504 PMC3318959 17898336

[B21] Foa E. B. Street G. P. (2001). Women and traumatic events. *J. Clin. Psychiatry* 62 29–34 11495093

[B22] Garrett J. E. Wellman C. L. (2009). Chronic stress effects on dendritic morphology in medial prefrontal cortex: sex differences and estrogen dependence. *Neuroscience* 162 195–207 10.1016/j.neuroscience.2009.04.057 19401219 PMC2720075

[B23] Goldstein J. M. Jerram M. Abbs B. Whitfield-Gabrieli S. Makris N. (2010). Sex differences in stress response circuitry activation dependent on female hormonal cycle. *J. Neurosci.* 30 431–438 10.1523/JNEUROSCI.3021-09.2010 20071507 PMC2827936

[B24] Hoover W. B. Vertes R. P. (2007). Anatomical analysis of afferent projections to the medial prefrontal cortex in the rat. *Brain Struct. Funct.* 212 149–179 10.1007/s00429-007-0150-4 17717690

[B25] Hurley K. M. Horst H. Moga M. M. Saper C. B. (1991). Efferent projections of the infralimbic cortex of the rat. *J. Comparat. Neurol.* 308 249–276 10.1002/cne.903080210 1716270

[B26] Izquierdo A. Wellman C. L. Holmes A. (2006). Brief uncontrollable stress causes dendritic retraction in infralimbic cortex and resistance to fear extinction in mice. *J. Neurosci.* 26 5733–5738 10.1523/JNEUROSCI.0474-06.2006 16723530 PMC6675270

[B27] Jinks A. L. McGregor I. S. (1997). Modulation of anxiety-related behaviours following lesions of the prelimbic or infralimbic cortex in the rat. *Brain Res.* 772 181–190 10.1016/S0006-8993(97)00810-X 9406971

[B28] Kendler K. S. Karkowski L. M. Prescott C. A. (1999). Causal relationships between stressful life events and the onset of major depression. *Am. J. Psychiatry* 156 837–841 10360120 10.1176/ajp.156.6.837

[B29] Kendler K. S. Kessler R. C. Walters E. E. MacLean C. Neale M. C. Heath A. C. (1995). Stressful life events, genetic liability, and onset of an episode of major depression in women. *Am. J. Psychiatry* 152 833–842 7755111 10.1176/ajp.152.6.833

[B30] Kessler R. C. (1997). The effects of stressful life events on depression. *Annu. Rev. Psychol.* 48 191–214 10.1146/annurev.psych.48.1.191 9046559

[B31] Koenigs M. Grafman J. (2009). Posttraumatic stress disorder: the role of medial prefrontal cortex and amygdala. *Neuroscientist* 15 540–548 10.1177/1073858409333072 19359671 PMC2771687

[B32] Kong L. Chen K. Womer F. Jiang W. Luo X. Driesen N. (2013). Sex differences of gray matter morphology in cortico-limbic-striatal neural system in major depressive disorder. *J. Psychiatr. Res*. 47 733–739 10.1016/j.jpsychires.2013.02.003 23453566 PMC3626116

[B33] Lebrón K. Milad M. R. Quirk G. J. (2004). Delayed recall of fear extinction in rats with lesions of ventral medial prefrontal cortex. *Learn. Mem.* 11 544–548 10.1101/lm.78604 15466306

[B34] Lebron-Milad K. Abbs B. Milad M. R. Linnman C. Rougemount-Bücking A. Zeidan M. A. (2012). Sex differences in the neurobiology of fear conditioning and extinction: a preliminary fMRI study of shared sex differences with stress-arousal circuitry. *Biol. Mood Anxiety Disord.* 2 1–10 10.1186/2045-5380-2-7 22738021 PMC3416700

[B35] LeDoux J. E. (2000). Emotion circuits in the brain. *Annu. Rev. Neurosci.* 23 155–184 10.1146/annurev.neuro.23.1.155 10845062

[B36] Liberzon I. Britton J. C. Phan K. L. (2003). Neural correlates of traumatic recall in posttraumatic stress disorder. *Stress* 6 151–156 10.1080/1025389031000136242 13129808

[B37] Likhtik E. Pelletier J. G. Paz R. Pare D. (2005). Prefrontal control of the amygdala. *J. Neurosci.* 25 7429–7437 10.1523/JNEUROSCI.2314-05.2005 16093394 PMC6725290

[B38] Lupien S. J. Lepage M. (2001). Stress, memory, and the hippocampus: can’ t live with it, can’ t live without it. *Behav. Brain Res.* 127 137–158 10.1016/S0166-4328(01)00361-8 11718889

[B39] Lupien S. J. McEwen B. S. Gunnar M. R. Heim C. (2009). Effects of stress throughout the lifespan on the brain, behaviour and cognition. *Nat. Rev. Neurosci.* 10 434–445 10.1038/nrn2639 19401723

[B40] Maeng L. Y. Shors T. J. (2012). Once a mother, always a mother: maternal experience protects females from the negative effects of stress on learning throughout their lifetime. *Behav. Neurosci.* 126 137–141 10.1037/a0026707 22181714 PMC3279153

[B41] Maeng L. Y. Waddell J. Shors T. J. (2010). The prefrontal cortex communicates with the amygdala to impair learning after acute stress in females but not in males. *J. Neurosci.* 30 16188–16196 10.1523/JNEUROSCI.2265-10.2010 21123565 PMC3073607

[B42] Majchrzak M Di Scala G. (2000). GABA and muscimol as reversible inactivation tools in learning and memory. *Neural Plast.* 7 19–29 10.1155/NP.2000.19 10709211 PMC2565374

[B43] Maroun M. Richter-Levin G. (2003). Exposure to acute stress blocks the induction of long-term potentiation of the amygdala – prefrontal cortex pathway in vivo. *J. Neurosci.* 23 4406–4409 12805280 10.1523/JNEUROSCI.23-11-04406.2003 PMC6740777

[B44] Martin J. H. (1991). Autoradiographic estimation of the extent of reversible inactivation produced by microinjection of lidocaine and muscimol in the rat. *Neurosci. Lett.* 127 160–164 10.1016/0304-3940(91)90784-Q 1881625

[B45] McDonald A. J. Mascagni F. Guo L. (1996). Projections of the medial and lateral prefrontal cortices to the amygdala: a *Phaseolus vulgaris* leucoagglutinin study in the rat. *Neuroscience* 71 55–75 10.1016/0306-4522(95)00417-3 8834392

[B46] McEwen B. S. (2005). Glucocorticoids, depression, and mood disorders: structural remodeling in the brain. *Metabolism* 54 20–23 10.1016/j.metabol.2005.01.008 15877308

[B47] Milad M. R. Pitman R. K. Ellis C. B. Gold A. L. Shin L. M. Lasko N. B. (2009). Neurobiological basis of failure to recall extinction memory in posttraumatic stress disorder. *Biol. Psychiatry* 66 1075–1082 10.1016/j.biopsych.2009.06.026 19748076 PMC2787650

[B48] Milad M. R. Quirk G. J. (2002). Neurons in medial prefrontal cortex signal memory for fear extinction. *Nature* 420 70–74 10.1038/nature01138 12422216

[B49] Nolen-Hoeksema S. (2012). Emotion regulation and psychopathology: the role of gender. *Annu. Rev. Clin. Psychol.* 8 161–187 10.1146/annurev-clinpsy-032511-143109 22035243

[B50] Nolen-Hoeksema S. Girgus J. S. (1994). The emergence of gender differences in depression during adolescence. *Psychol. Bull.* 115 424–443 10.1037/0033-2909.115.3.424 8016286

[B51] O’Donnell M. L. Creamer M. Pattison P. (2004). Posttraumatic stress disorder and depression following trauma: understanding comorbidity. *Am. J. Psychiatry* 161 1390–1396 10.1176/appi.ajp.161.8.1390 15285964

[B52] Paxinos G. Watson C. (1997). *The Rat Brain in Stereotaxic Coordinates*. 3rd Edn. Orlando: Academic Press

[B53] Pendyam S. Bravo-Rivera C. Burgos-Robles A. Sotres-Bayon F. Quirk G. J. Nair S. S. (2013). Fear signaling in the prelimbic-amygdala circuit: a computational modeling and recording study. *J. Neurophysiol.* 110 844–861 10.1152/jn.00961.2012 23699055 PMC3742978

[B54] Quirk G. J. Likhtik E. Pelletier J. G Paré D. (2003). Stimulation of medial prefrontal cortex decreases the responsiveness of central amygdala output neurons. *J. Neurosci.* 23 8800–8807 14507980 10.1523/JNEUROSCI.23-25-08800.2003 PMC6740415

[B55] Radley J. J. Arias C. M. Sawchenko P. E. (2006). Regional differentiation of the medial prefrontal cortex in regulating adaptive responses to acute emotional stress. *J. Neurosci.* 26 12967–12976 10.1523/JNEUROSCI.4297-06.2006 17167086 PMC6674963

[B56] Rauch S. L. Shin L. M. Phelps E. A. (2006). Neurocircuitry models of posttraumatic stress disorder and extinction: human neuroimaging research – past, present, and future. *Biol. Psychiatry* 60 376–382 10.1016/j.biopsych.2006.06.004 16919525

[B57] Rosenkranz J. A. Grace A. A. (2001). Dopamine attenuates prefrontal cortical suppression of sensory inputs to the basolateral amygdala of rats. *J. Neurosci.* 21 4090–4103 11356897 10.1523/JNEUROSCI.21-11-04090.2001 PMC6762693

[B58] Shansky R. M. Hamo C. Hof P. R. Lou W. McEwen B. S. Morrison J. H. (2010). Estrogen promotes stress sensitivity in a prefrontal cortex – amygdala pathway. *Cereb. Cortex* 20 2560–2567 10.1093/cercor/bhq003 20139149 PMC2951843

[B59] Shin L. M. Wright C. I. Cannistraro P. A. Wedig M. M. McMullin K. Martis B. (2005). A functional magnetic resonance imaging study of amygdala and medial prefrontal cortex responses to overtly presented fearful faces in posttraumatic stress disorder. *Arch. Gen. Psychiatry* 62 273–281 10.1001/archpsyc.62.3.273 15753240

[B60] Shin L. M. Liberzon I. (2010). The neurocircuitry of fear, stress, and anxiety disorders. *Neuropsychopharmacology* 35 169–191 10.1038/npp.2009.83 19625997 PMC3055419

[B61] Shors T. J. (2004). Learning during stressful times. *Learn. Mem.* 11 137–144 10.1101/lm.66604 15054128 PMC3364672

[B62] Shors T. J. Chua C. Falduto J. (2001). Sex differences and opposite effects of stress on dendritic spine density in the male versus female hippocampus. *J. Neurosci.* 21 6292–6297 11487652 10.1523/JNEUROSCI.21-16-06292.2001 PMC6763131

[B63] Shors T. J. Lewczyk C. Pacynski M. Mathew P. R. Pickett J. (1998). Stages of estrous mediate the stress-induced impairment of associative learning in the female rat. *Neuroreport* 9 419–423 10.1097/00001756-199802160-00012 9512383

[B64] Sierra-Mercado D. Padilla-Coreano N. Quirk G. J. (2011). Dissociable roles of prelimbic and infralimbic cortices, ventral hippocampus, and basolateral amygdala in the expression and extinction of conditioned fear. *Neuropsychopharmacology* 36 529–538 10.1038/npp.2010.184 20962768 PMC3005957

[B65] Sotres-Bayon F. Bush D. E. LeDoux J. E. (2004). Emotional perseveration: an update on prefrontal-amygdala interactions in fear extinction. *Learn. Mem.* 11 525–535 10.1101/lm.79504 15466303

[B66] Sotres-Bayon F. Sierra-Mercado D. Pardilla-Delgado E. Quirk G. J. (2012). Gating of fear in prelimbic cortex by hippocampal and amygdala inputs. *Neuron* 4 804–812 10.1016/j.neuron.2012.09.028 23177964 PMC3508462

[B67] Tang Y. Kong L. Wu F. Womer F. Jiang W. Cao Y. (2012). Decreased functional connectivity between the amygdala and the left ventral prefrontal cortex in treatment-naive patients with major depressive disorder: a resting-state functional magnetic resonance imaging study. *Psychol. Med.* 43 1921–1927 10.1017/S0033291712002759 23194671

[B68] Tolin D. F. Foa E. B. (2006). Sex differences in trauma and posttraumatic stress disorder: a quantitative review of 25 years of research. *Psychol. Bull.* 132 959–992 10.1037/0033-2909.132.6.959 17073529

[B69] Vertes R. P. (2004). Differential projections of the infralimbic and prelimbic cortex in the rat. *Synapse* 51 32–58 10.1002/syn.10279 14579424

[B70] Vidal-Gonzalez I. Vidal-Gonzalez B. Rauch S. L. Quirk G. J. (2006). Microstimulation reveals opposing influences of prelimbic and infralimbic cortex on the expression of conditioned fear. *Learn. Mem.* 13 728–733 10.1101/lm.306106 17142302 PMC1783626

[B71] Waddell J. Bangasser D. A. Shors T. J. (2008). The basolateral nucleus of the amygdala is necessary to induce the opposing effects of stressful experience on learning in males and females. *J. Neurosci.* 28 5290–5294 10.1523/JNEUROSCI.1129-08.2008 18480285 PMC2680275

[B72] Wang L. Dai Z. Peng H. Tan L. Ding Y. He Z. (2013). Overlapping and segregated resting-state functional connectivity in patients with major depressive disorder with and without childhood neglect. *Hum. Brain Mapp.* 10.1002/hbm.22241 [Epub ahead of print] PMC6869506 23408420

[B73] Wood G. E. Beylin A. V. Shors T. J. (2001). The contribution of adrenal and reproductive hormones to the opposing effects of stress on trace conditioning in males versus females. *Behav. Neurosci.* 115 175–187 10.1037/0735-7044.115.1.175 11256441

[B74] Wood G. E. Shors T. J. (1998). Stress facilitates classical conditioning in males, but impairs classical conditioning in females through activational effects of ovarian hormones. *Proc. Natl. Acad. Sci. U.S.A.* 95 4066–4071 10.1073/pnas.95.7.4066 9520494 PMC19964

[B75] Yehuda R. (2002). Post-traumatic stress disorder. *N. Engl. J. Med.* 346 108–114 10.1056/NEJMra012941 11784878

